# Long-Term Risk of Progression From Unilateral to Bilateral Méniere's Disease: A Systematic Review and Meta-Analysis

**DOI:** 10.1097/MAO.0000000000004491

**Published:** 2025-04-11

**Authors:** Thomas James Hudson, Linette Shu Hwei Tan, Veronica Phillips, Jameel Muzaffar, Manohar Bance

**Affiliations:** ∗ Department of Otolaryngology–Head and Neck Surgery, Cambridge University Hospitals NHS Foundation Trust, Cambridge, UK; † Medical Library, School of Clinical Medicine, University of Cambridge, Cambridge, UK; ‡ Department of Ear, Nose and Throat Surgery, University Hospitals Birmingham NHS Foundation Trust, Birmingham, UK; § Department of Clinical Neurosciences, University of Cambridge, Cambridge, UK

**Keywords:** Aural vertigo, Bilateral, Endolymphatic hydrops, Intratympanic, Menière, Metachronous, Otogenic vertigo

## Abstract

**Objective:**

To evaluate the overall risk of conversion from unilateral to bilateral Méniere's disease (MD), the time interval from initial diagnosis to conversion, and any risk factors or audiometric trends associated with this process.

**Databases Reviewed:**

Medline, Embase, Cochrane Library, Scopus, Web of Science, and ClinicalTrials.Gov.

**Methods:**

This review was reported according to the Preferred Reporting Items for Systematic Reviews and Meta-Analyses (PRISMA) guidelines. Studies reporting longitudinal progression of unilateral to bilateral MD were included. Random-effects meta-analyses were performed to evaluate the proportion of cases that converted and the mean time to conversion, and a narrative synthesis described risk factors and audiometric data.

**Results:**

A total of 11 studies met the inclusion criteria. For overall conversion risk, meta-analysis of the 9 relevant studies (n = 1583) yielded a risk of 13% (95% CI, 12–15%). Mean time to conversion was 8.2 years (95% CI, 5.9–10.6; *I*^2^ = 46%), and a combined Kaplan-Meier analysis revealed a prolonged distribution of conversions including 10% that converted 20 years or later after initial diagnosis. Risk factors and audiometric data were sparsely reported, but there may be a correlation between conversion risk and first-sided ear surgery (protective), history of tympanic membrane perforation, and baseline hearing loss in the unaffected ear.

**Conclusion:**

There is a significant risk of conversion from unilateral to bilateral MD that must be taken into account when considering ablative treatment options, even late into its course. Further work will be required to better characterize risk/protective factors and audiometric trends.

## BACKGROUND

Méniere's disease (MD) is a debilitating disorder of the inner ear characterized by episodic vertigo, hearing loss, tinnitus, and aural fullness. The hallmark histopathologic feature, endolymphatic hydrops, has long been established in experimental and clinical study (1–4). However, despite over one-and-a-half centuries and a wealth of literature since Prosper Méniere first attempted to describe this disorder (5), there still remains much that is not fully understood about the pathophysiology, management, and clinical course of this disease (6).

Contemporary diagnosis of MD is based on clinical assessment alone, and the most recent set of criteria was established by international committee initially published by the Barany Society in 2015 and later agreed upon by the American Academy of Otolaryngology (AAO) (7,8). Objective tests, such as delayed post-gadolinium magnetic resonance imaging (MRI) (9) and electrocochleography (10), are not essential but have been shown to be useful adjuncts in diagnosis and guiding management. MD can be a unilateral (UMD) or bilateral (BMD) process, and bilateral cases can be subclassified into synchronous or metachronous based on the onset of each ear. Additional classification systems have been described, separating patients based on features including migraine status, family history, autoimmune disease, and presence of delayed endolymphatic hydrops (11,12).

Treatment of MD typically proceeds in a stepwise approach, seeking a regimen that balances effective vertigo prophylaxis against side effects, importance and quality of remaining hearing, the contralateral ear's status, and patient preferences (13,14). Although some patients can be managed with diet and lifestyle modifications alone, first-line medications are commonly given and include diuretics, with some low level of evidence supporting their use (15), and Betahistine, with mixed evidence that includes multiple randomized control trials failing to show superiority over placebo (16,17). When medications fail, management then typically turns to preserving procedures, with intratympanic corticosteroids being commonly used, with some proven efficacy (18). This can be followed by endolymphatic sac surgery, although again, its use is controversial with previous placebo-controlled studies failing to show benefit (19), or vestibular nerve section, which sacrifices vestibular function and carries higher risks of serious complications as it is an intracranial procedure (20). Finally, when the above fail or when hearing becomes nonfunctional, management then turns to ablative approaches. Intratympanic gentamicin has proven efficacy along with generally low risks of hearing loss, but with vestibular sacrifice (21), triple semicircular canal occlusion surgery has early evidence demonstrating vertigo control along with some preserved hearing and otolithic function (22), and surgical labyrinthectomy, which is generally reserved for the most severe and refractory cases.

Management of bilateral Méniere's disease can therefore be a challenge in many cases given that some of the most potent treatment options available can eliminate residual hearing and balance. Similarly, given that a portion of patients can convert from unilateral to bilateral later in the course of the disease, considering any ablative option must also take into account the risks of progression and future bilateral deafness, even with a seemingly normal contralateral ear. At the moment though, there is a lack of robust data that describes the overall risk of bilateral conversion, the time interval between ears, and whether there are any factors that could be used for risk estimation. The objectives of this review will therefore be to:

- evaluate the overall risk of a given patient diagnosed with unilateral MD converting to bilateral MD based on longitudinal data from the literature,
- establish the distribution of time intervals expected from first ear to second,
- determine clinical risk factors (if any) that could help predict conversion, and
- assess the degree of hearing loss in the first and second ears.

## METHODS

### Literature Search

This review was reported according to the Preferred Reporting Items for Systematic Reviews and Meta-Analyses (PRISMA) 2020 guidelines (23), and was registered prospectively on PROSPERO (ID: CRD42024541421). The completed PRISMA checklist is available in Supplemental File 1, http://links.lww.com/MAO/C84.

We conducted a systematic literature search using the databases Medline (via Ovid), Embase (via Ovid), Cochrane Library, Web of Science (Core Collection), and Scopus from inception to May 2024 using the MeSH and key search terms: Méniere, endolymphatic hydrops, auditory vertigo, aural vertigo, otogenic vertigo, bilateral (see supplementary information for full search criteria). The search strategy was designed by a search specialist (V.P.) and is included in Supplemental File 2, http://links.lww.com/MAO/C85. It was peer-reviewed by two librarian colleagues using the Peer Review of Electronic Search Strategies (PRESS) checklist (24) and evaluated against the PRISMA-S guidelines (25). Databases were searched separately rather than multiple databases being searched on the same platform (V.P.). The search syntax was adapted for each database, accounting for variation between thesaurus terms/controlled vocabulary. Results were imported to Endnote 21 for deduplication, using the method outlined by Bramer et al. (26) In addition, the ClinicalTrials.gov website was searched from inception to May 2024 to identify ongoing or unpublished clinical trials. The search was repeated at the end of analysis in November 2024, and additional articles were included when appropriate.

Titles and abstracts were imported into the Rayyan systematic review software (www.rayyan.ai) (27), and then screened for relevance by two independent reviewers (L.T. and T.H.). Two reviewers (L.T. and T.H.) independently evaluated full texts for inclusion using predetermined eligibility criteria. Bibliographies of included studies were searched for additional relevant studies. Discrepancies were resolved through consensus.

### Inclusion and Exclusion Criteria

PICOTS eligibility criteria were used:

Population/Exposure: Adults with a diagnosis of unilateral Méniere's Disease based on established diagnostic criteria.

Comparator(s)/control: None.

Outcome(s): Conversion from unilateral to metachronous bilateral MD.

**Time Course:** Any point during the study duration after an initial diagnosis of unilateral disease.

**Study Design:** Any peer-reviewed study reporting longitudinal data on a cohort of patients that developed metachronous BMD, with or without UMD-only controls.

Exclusion criteria:

1. Animal or cadaveric study
2. Pharmacological models
3. Endolymphatic hydrops without a diagnosis of MD
4. Non-English language studies
5. Opinion or editorial articles
6. Full text unavailable
7. Absent or insufficient metachronous (longitudinal) data
8. Nonstandard or unspecified diagnostic criteria for MD

### Data Extraction

Two reviewers (T.H. and L.T.) independently extracted data from included studies using pre-established computerized spreadsheets piloted during the preliminary literature search, which were compared to ensure accuracy. Inconsistencies were resolved through consensus. Trial characteristics, patient demographics, age of onset of MD, conversion to bilateral status, time to onset of disease on second side, duration of follow-up, risk and protective factors, and degree of hearing loss on audiometric testing were recorded by preference where available. Where figure resolution permitted accurate extraction, graphical data were used.

### Risk of Bias

After the literature search was completed, it was determined that this review would be based solely on nonrandomized observational studies. The risk of bias assessment was therefore performed exclusively using the Risk Of Bias In Non-randomized Studies of Exposure (ROBINS-E) tool (28), and visualized using robvis (29). All seven categories and overall risk were evaluated for each included study with respect to the bias they introduce in answering the current study question and objectives.

### Data Synthesis

All statistical analysis was completed in R v4.3.0 (R Foundation for Statistical Computing, Vienna, Austria), primarily using the “meta” and “metafor” packages. To evaluate the overall risk of converting from unilateral to bilateral MD, a meta-analysis of proportions was performed using a generalized linear mixed-effects model (GLMM) with logit-transformed proportions (30,31). A funnel plot was created, and Egger test (32) performed to characterize the statistical risk of bias, and *I*^2^ test of heterogeneity was performed and included with a forest plot. Upon completion of data collection, it was noted that a subset of the included studies selected for a biased sample of MD patients (e.g., exclusively patients who received intratympanic gentamycin, who received labyrinthectomy, who had abnormal imaging findings, or who had intractable disease). A second analysis was performed excluding these studies.

For the time interval to second side, data were first extracted from the published Kaplan-Meier curves in three studies to establish individual patient data, and a combined Kaplan-Meier curve was produced. For studies that reported a statistical distribution, Egger test was performed and a meta-analysis of the mean time to conversion was then produced using a restricted maximum likelihood (REML) random-effects model (33). Finally, given the scarcity of data describing risk factors and audiometry, a narrative synthesis alone was produced.

## RESULTS

### Search Results and Characteristics

**FIG. 1:**
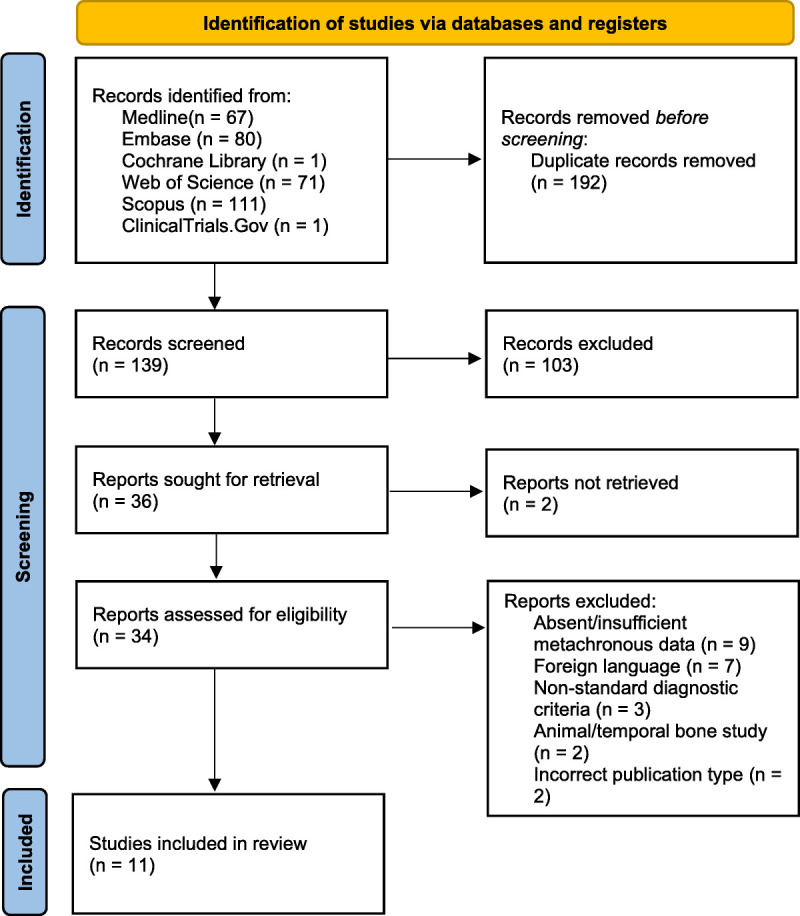
PRISMA flow diagram for study selection (23). Figure 1 is permitted according to the Creative Commons Attribution (CC BY 4.0):https://creativecommons.org/licenses/by/4.0/https://www.prisma-statement.org/prisma-2020-flow-diagram.

**FIG. 2:**
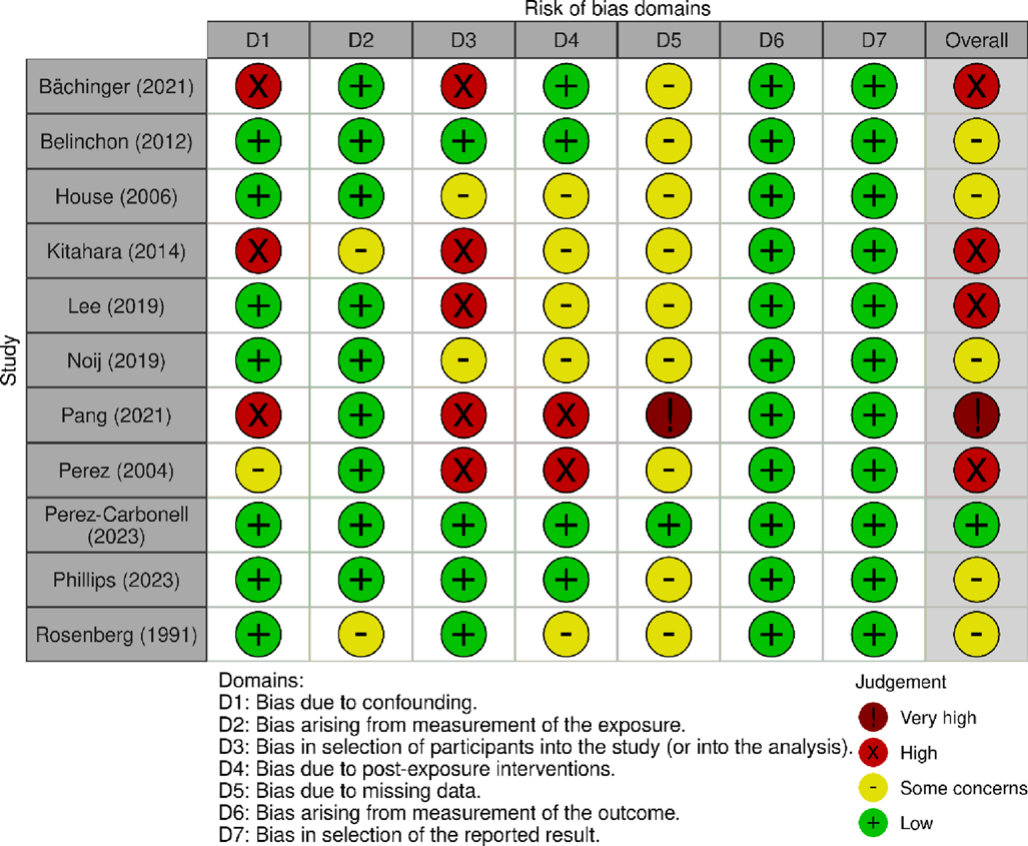
Risk of bias assessment based on the ROBINS-E tool (28) (plot produced using robvis (29)). Figure 2 is permitted according to the Creative Commons Attribution-NonCommercial-NoDerivatives 4.0 International License:https://creativecommons.org/licenses/by-nc-nd/4.0/deed.enhttps://www.riskofbias.info/welcome.

The literature search produced 139 unique articles, of which title and abstract screening excluded 103. Of the remaining 36, 2 articles were unable to be retrieved, and the remaining 34 were subject to a full-text review. After applying the appropriate selection criteria, 11 articles remained for analysis (see Fig. 1 for a detailed flow diagram (23)). Critical appraisal of the included articles revealed that the majority had either some concerns or were at a high risk of bias (Figure 2 (28,29)). Most studies had some concerns due to potential missing data. This occurred because of their retrospective observational study design, where it was not possible to guarantee complete long-term data for patients who did not appear to undergo bilateral conversion (i.e., censored data, short follow-up time). Four studies had a high risk of bias, caused by skewed selection criteria as mentioned above (i.e., only patients who received a surgical or chemical labyrinthectomy, who had intractable disease, or who had abnormal imaging findings).

Table 1 details the descriptive characteristics of each of the included studies. Note that, although not all studies used the 2015 AAO/BS criteria (7,8) for diagnosis, it was deemed that the 1995 and 1985 AAO criteria (34,45) would be adequate given their similar requirements of multiple vertigo episodes, fluctuating sensorineural hearing loss, and aural symptoms.

**TABLE 1:** Study characteristics

Study	Study Design	Diagnostic Criteria	No. Cases Initially Diagnosed with UMD	Definition of Bilateral Disease	Mean Age of Initial Disease Onset	Length of Follow-Up	Clinical Risk or Protective Factors Evaluated	Audiometry Data Reported
Bächinger (2021) (34) *^a^*	R-Co	2015 AAO/BS	44	First ear: def. MD, Second ear: V + HL	Tot: 39.0 yr	Max: 31 yr	None	None
Belinchon (2012) (35)	P-Co	1995 AAO	230	First ear: def. MD, Second ear: NS	NS	Max: 31 yr	None	None
House (2006) (36)	R-Co	2015 AAO/BS	165	First ear: MD- NOS, [e_k] Second ear: HL + T/A	Uni: 51.8 yr, Met: 47.2 yr	Uni M: 8.4 yr, Met M: 10.3 yr, Max: 45 yr	Allergies, surgery	*t:* Presentation, *s:* Both
Kitahara (2014) (37) *^a^*	P-Co	1995 AAO	237	First ear: Intractable def. MD, Second ear: V + HL/T/A	Tot: 48.7 yr	5 yr fixed	Surgery	*t:* Preoperative, *s:* NS
Lee (2019) (38) ^b^	R-Co	2015 AAO/BS	16	First ear: def. MD, Second ear: NS	Met: 41.7 yr	Mdn: 8 yr, IQR: 5.8–12.75	FHx, migraine	*t:* Most recent, *s:* Both
Noij (2019) (39)	R-Co	2015 AAO/BS	49	First ear: MD-NOS, [e_k] Second ear: Def. or Prob. MD	Tot: 49.2 yr	Uni M: 14.7 yr, Range: 5– 30, [e_k] Met M: 11.3 yr, Range: 2–24	None	None
Pang (2021) (40) *^a^*	R-Co	1995 AAO	84	First ear: MD-NOS, [e_k] second ear: Any HL/V/A/T	NS	Uni M: 1.7 yr after surgery	Surgery	None
Perez (2004) (41) *_a_*	R-Co	1995 AAO	101	Both ears: MD-NOS	Tot: 42 yr	At 2 yr: 101 remained, 5 yr: 96, 10 yr: 61,20 yr: 14	None	None for Met. MD
Pérez-Carbonell (2022) (42) **	R-Co	2015 AAO/BS	45	First ear: MD- NOS, [e_k] Second ear: HL + T	Tot: 50.1 yr	Range: 1–34 yr	Migraine, age, sex^c^	*t:* NS, *s:* NS
Philips (2023) (43)	R-Co	2015 AAO/BS	275	First ear: def. or prob. MD, Second ear: HL	NS	Range: 1–29 yr	Psoriasis, ear infection, depression, history of perforation	None
Rosenberg (1991) (44)	R-Co	1985 AAO	398	First ear: MD- Second ear: NS	Tot: 49 yr	NS	Surgery	None

*^a^* Studies where the inclusion criteria selected for a sample not representative of the general population of Méniere*'*s disease patients (*i.e.,* exclusively those managed surgically or by chemical labyrinthectomy, those with intractable disease, or those with abnormal imaging).

*^b^* Studies evaluating bilateral cases with no unilateral controls.

*^c^* A nested case-control study was performed separate to the main analysis, evaluating risk factors of metachronous MD versus unilateral.

A indicates aural fullness; AAO, American Association of Otolaryngology; BS, Barany Society; Co, cohort; def, definite; FHx, family history; HL, hearing loss; M, mean; MD, Méniere*'*s disease; Mdn, median; Met, metachronous; NOS, not otherwise specified; NS, not specified; p, prospective; prob, probable; R, retrospective; s, side; SD, standard deviation; t, time; T, tinnitus; Tot, total population; Uni, unilateral; V, vertigo.

### Overall Risk of Bilateral Conversion

**FIG. 3:**
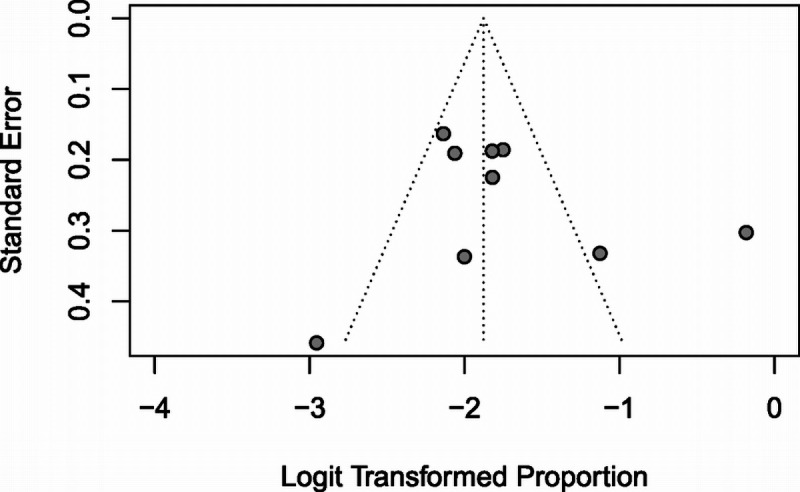
Funnel plot of included studies based on the proportions of unilateral patients converted bilateral.

Of the 11 studies, 9 reported samples that included both unilateral and metachronous bilateral disease cases, which could therefore be included in a proportion analysis. These studies were compiled into the funnel plot seen in Figure 3, where one study (35) was shown to be grossly outside the expected statistical proportions, two studies (36,37) were borderline outside, and the other six appeared appropriate without any clear asymmetry. Egger test confirmed no statistically significant asymmetry (*p* = 0.51).

**FIG. 4:**
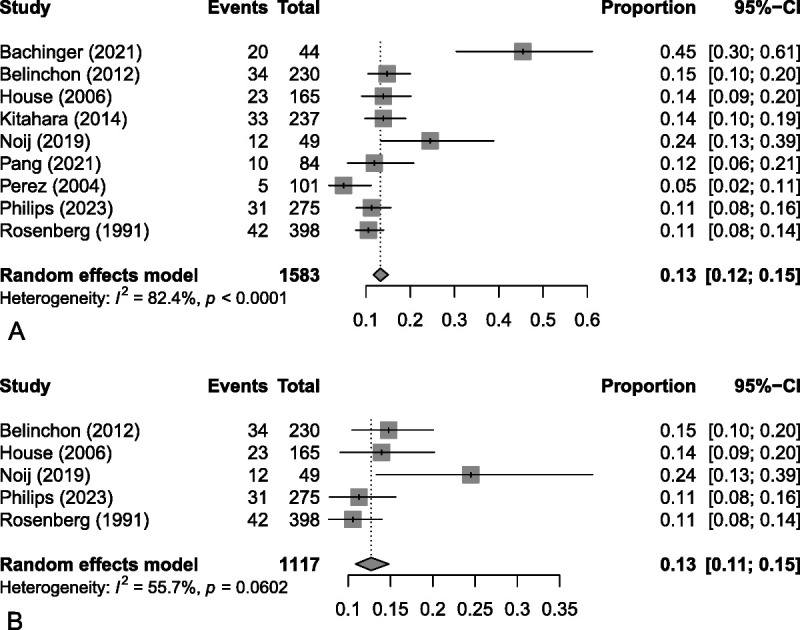
Forest plots demonstrating the meta-analysis of proportions, evaluating conversion from unilateral to metachronous bilateral Méniere's disease. *A*, Complete analysis, *B*, Post-hoc analysis excluding studies with a high risk of bias caused by skewed inclusion criteria.

Overall, including these nine studies resulted in a total of 1,583 patients with an initial diagnosis of unilateral MD, and 210 that later converted during their respective study periods. Meta-analysis of these studies revealed an overall rate of conversion to be 13% (95% CI, 12–15%) (see Fig. 4A). Note that the *I*^2^ test revealed a substantial amount of heterogeneity (*I*^2^ = 82%). As previously mentioned, there was concern during data collection regarding the inclusion criteria of four studies that skewed away from a typical sample of MD patients. A post-hoc meta-analysis was then performed excluding these articles (Fig. 4B). This resulted in a rate of conversion of 13% (95% CI, 11–15%), which was quite similar to the previous calculation but with a moderate degree of heterogeneity (*I*^2^ = 56%) that did not reach significance (*p* = 0.06).

### Time to Conversion

**FIG. 5:**
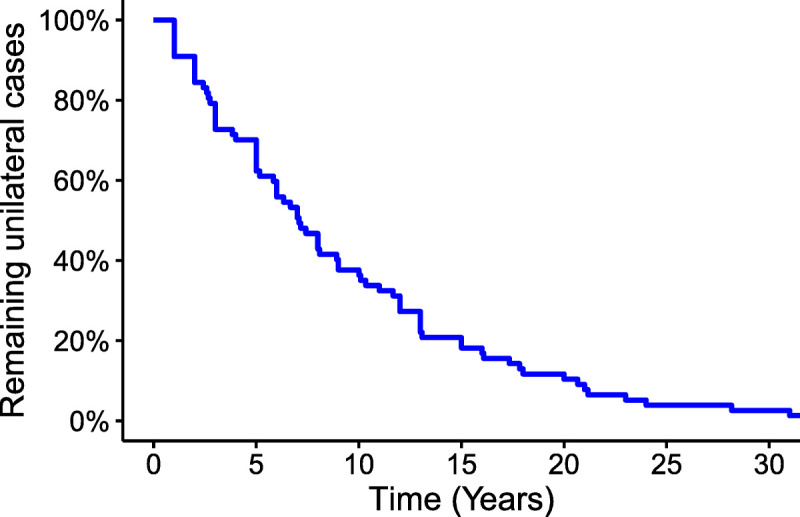
Combined Kaplan-Meier curve illustrating time to conversion, based on metachronous MD data from three studies (35,36,42).

**FIG. 6:**
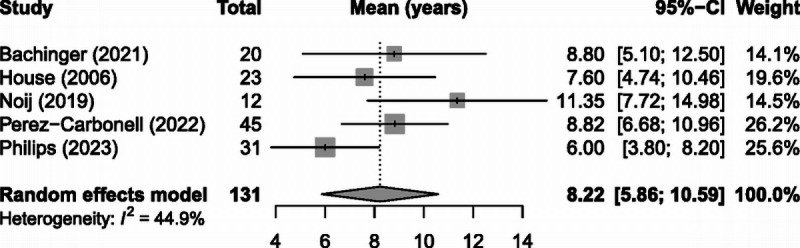
Meta-analysis of mean time to conversion.

During data collection, it was noted that three studies provided Kaplan-Meier curves demonstrating time to conversion. After extracting the individual patient data, a combination Kaplan-Meier curve was then created illustrating the overall time to conversion (Fig. 5). These data suggest an overall median of 7.08 years, with 5-, 10-, and 20-year remaining proportions of 62, 36, and 10%, respectively. The latest case of conversion in these data were 31 years, whereas the latest case across all studies was 45 years. For mean time to conversion, five studies provided statistically adequate data, and meta-analysis revealed a mean conversion time of 8.22 years (95% CI, 5.9–10.6) (Fig. 6). This demonstrated a modest degree of heterogeneity (*I*^2^ = 45%) that did not reach significance, and Egger test additionally demonstrated no significant statistical bias (*p* = 0.31).

### Risk and Protective Factors

Several studies evaluated risk or protective factors for conversion from UMD to metachronous BMD. The most frequently reported of these was having a surgery performed on the first ear. Three studies evaluated this retrospectively, with the first (38) showing a significant protective effect where only 5.9% of patients surgically treated (vestibular nerve section, endolymphatic surgery, cochleosacculotomy) converted compared to 17% of medically treated (*p* < 0.01). The second (39), though, suggested that there was no difference among their surgical and nonsurgical subgroups (no quantities or types of surgery reported), and the third (40), evaluating only patients who received surgical labyrinthectomy, suggested an overall conversion rate of 12%. There was one nonrandomized prospective trial (41), which demonstrated a significant protective effect of endolymphatic surgery in the subgroup of patients who had evidence of preoperative contralateral hydrops based on glycerol test and electrocochleography (*p* = 0.022), but not the subgroup with normal contralateral testing (*p* = 0.231).

Two studies evaluated migraine as a risk factor, where the first (42) suggested a correlation with increased risk of conversion compared to unilateral controls, but without reaching significance (RR = 2.88; 95% CI, 0.58–14.2). The second (43) did not evaluate against unilateral controls, but instead analyzed whether migraine was a risk factor for early versus late conversion, which did not reach significance (*p* = 0.32). Psoriasis, history of ear infections, and depression were all evaluated as risk factors in one study (44), which found each to be independently significant (*p* = 0.003, 0.001, 0.047, respectively) but did so comparing UMD to all BMD cases (including synchronous). That same study evaluated a previous or current history of tympanic membrane perforation (side not specified), appropriately comparing only metachronous BMD to UMD controls, and found a significant relationship (OR, 3.84; 95% CI, 1.30–11.38). Allergy, family history, age, sex, and number of symptoms at initial presentation were all evaluated individually and not found to be significant.

### Audiometry

Audiometric results were sparse and not uniformly reported among included studies. Of the four studies providing data, the first (39) compared pure-tone averages in affected and non-affected ears at presentation. When evaluating the initially unaffected ear, they found a significantly higher threshold in those who later converted to bilateral (initial PTA = 19.1 dB) compared to those that remained unilateral (initial PTA = 14.5 dB) (*p* < 0.02). The second (41) study provided pre-operative (if applicable) worst-recorded hearing levels. Thresholds appeared higher among those with evidence of silent contralateral endolymphatic hydrops (+EH 60.8 dB versus −EH 52.2 dB and + EH 56.3 dB versus −EH 47.5 dB in operative and nonoperative cases, respectively), but there was no information on which ear was tested, what hearing metric was used, or statistical correlation. The third (42) similarly did not specify side, timing, or type of test, but suggested that low-frequency hearing levels were worse in synchronous compared to metachronous cases (28.35 versus 36.71 dB, *p* = 0.049). The fourth (43) of these studies reported the most recent pure-tone average results, where there was a trend suggesting that the first and second involved ears were worse in metachronous (69.4 and 63.1 dB, respectively) compared to synchronous (52.3 and 43.9 dB), but this was not significant (*p* = 0.052).

## DISCUSSION

Management of severe unilateral Méniere's disease can be a significant clinical challenge. Some of the most effective treatment options available, such as intratympanic gentamicin and surgical labyrinthectomy, ablate residual hearing and balance function in the affected ear. Given the chance of later converting to bilateral disease and developing non-serviceable hearing or vestibular loss in the contralateral ear, care must be taken in assessing the risks and accurately counseling the patient before proceeding. This review aimed to provide some clarity in this risk assessment. More specifically, it examined longitudinal data of patients with an initial diagnosis of unilateral MD to determine the overall risk of converting to bilateral over time. It additionally evaluated the anticipated time to conversion, risk factors for predicting conversion, and audiometric data over time of this patient group.

Overall risk of conversion was demonstrated to be 13% (95% CI, 12–15%) based on a combined total of 1,583 patients. This analysis yielded a significant amount of statistical heterogeneity (*I*^2^ = 82%), and a post-hoc analysis excluding significantly biased studies demonstrated a very similar risk of 13% (95% CI, 11–15%) but with moderate heterogeneity (*I*^2^ = 56%). There were several other sources of heterogeneity evident in the study characteristics. An important one arose from the variable definition of contralateral involvement, where diagnostic criteria of MD in the second ear ranged from having hearing loss and aural symptoms alone up to requiring a formal diagnosis of definite MD with the typical low-frequency hearing loss and distinct new vertigo episodes. There currently are no standardized criteria for establishing the diagnosis of metachronous BMD, and although most included studies seemed to agree that active vertigo episodes should be a component, at least one argued that there is no way to be certain vertigo is arising from the new ear and therefore should not be required (39). Another important source of heterogeneity arose from the length of follow-up. Given the observational nature of the included studies and the prolonged interval that can occur in between ears (maximum 45 yr among included studies), there is a possibility that cases of late conversion were missed, which would have been variable given the range of follow-up times recorded.

Mean time to conversion was demonstrated to be 8.22 years (95% CI, 5.86–10.59). This analysis was less prone to heterogeneity (*I*^2^ = 45%), which may be due to it focusing solely on patients who converted to bilateral, and therefore was not subject to bias arising from variable follow-up duration. The combined Kaplan-Meier curve (Fig. 5) illustrated the distribution of conversions over time, where a significant proportion of patients developed contralateral disease late into the follow-up period (36% converted later than 10 yr, 10% later than 20 yr). This refutes the previously described notion that there is a safe number of years, beyond which conversion is unlikely (46). Indeed, given that the most intense portion of the disease tends to occur within the first decade from diagnosis (47), and that the majority of ablative treatments appear to be given within the first one to two decades (37,38), it would be prudent to always caution patients with unilateral disease about the risk of contralateral involvement when offering these options.

The sparsity of data makes it difficult to draw any robust conclusions regarding risk/protective factors or audiometric patterns. Although there was some evidence to suggest that having surgery performed in UMD had a protective effect against conversion, this was not consistently demonstrated. There was only one prospective analysis (41) of this, which suggested that it may only be significant in patients with contralateral silent endolymphatic hydrops, but their subgroups with this finding were relatively small (35 operated and 18 non-operated). There also is a lack of clarity on the pathophysiologic connection between ipsilateral surgery and contralateral disease progression, where some previous study has suggested that it may be related to changes in systemic antidiuretic hormone levels (48–50) but without confirmatory clinical data. Presence of psoriasis, depression, ear infections, and history of perforation were all statistically significant as risk factors in a single retrospective analysis, whereas some of the more commonly implicated risk factors in Méniere's disease such as family history, age, and migraine did not yield significance. Further study would be required to assess these. In terms of audiometric results, one study (39) demonstrated a potentially relevant correlation where the normal contralateral ear had higher initial thresholds in patients who later converted to bilateral. Otherwise, hearing data were not clearly or consistently reported, and characteristics like the progression of hearing loss over time remain uncertain.

There were several important limitations in the current study, some of which have been detailed above. In the review process, a portion of excluded studies were in a different language (mostly Japanese), some of which were suspected to be relevant based on a translated abstract. Only two reviewers from the same center went through abstracts, articles, and data collection, which may have introduced bias from having a similar approach to and understanding of MD. Among the included articles, heterogeneity between included studies was a significant limitation, and aside from the already mentioned inconsistency in the definitions of contralateral involvement, lengths of follow-up, and initial study inclusion criteria, there additionally was heterogeneity in the treatments offered, methods of data collection (retrospective chart review, survey, prospective analysis), and years of publication (spanning over 30 yr). Studies were largely retrospective, which may have introduced factors like recall and response bias, and there were no studies providing a higher level of evidence. None of the included articles provided comprehensive lists of demographic information, and it is impossible to assess for bias arising from features like socioeconomic status or comorbidities. Beyond this, included studies were carried out in seven different countries, which could have introduced bias from differences in health care systems (i.e., public versus private) or cultural values.

## CONCLUSION

This was the first systematic review that characterized the conversion of unilateral to metachronous bilateral Méniere's disease. Meta-analysis based on longitudinal data yielded an overall conversion rate of 13% (95% CI, 12–15%). The mean time interval between ears was 8.22 years (95% CI, 5.9–10.6), and a combined Kaplan-Meier analysis revealed that a significant proportion of conversions happened late in the disease course (over 10% happened later than 20 yr from the initial diagnosis). There were insufficient consistent data to establish any clear risk/protective factors or audiometric trends. Future work could further characterize the correlation between conversion rate with ipsilateral otologic surgery and history of tympanic membrane perforation and could assess the trend of hearing loss in each ear over time.
